# Multiplexed Fiber-Optic Fluorescence for Functional Monitoring of Perfused Hearts

**DOI:** 10.1021/acs.analchem.5c03270

**Published:** 2025-10-22

**Authors:** Jianrong Qiu, Edward Waters, Emily Lupton, Friedrich Baark, Antoine L. D. Wallabregue, Stuart J. Conway, Richard Southworth, Mads S. Bergholt

**Affiliations:** † Centre for Craniofacial and Regenerative Biology, 4616 King’s College London, London SE1 9RT, U.K.; ‡ School of Biomedical Engineering & Imaging Sciences, King’s College London, King’s Health Partners, St Thomas’ Hospital, London SE1 7EH, U.K.; § Department of Chemistry, 6396 University of Oxford, Oxford OX1 3TA, U.K.; ∥ Department of Chemistry & Biochemistry, 8783 UCLA, Los Angeles, California 90095-1569, United States

## Abstract

Monitoring molecular dynamics in the heart is essential for advancing our understanding of cardiac physiology and biochemistry in both healthy and diseased states, as well as for guiding the development and evaluation of novel cardiac therapies. We present a multiexcitation, ratiometric fiber-optic spectroscopic platform for noninvasive, real-time monitoring of biochemical and physiological processes in isolated Langendorff-perfused rat hearts. The system employs a fiber-optic balloon probe capable of concurrent optical measurements and intraventricular pressure sensing, thereby providing complementary physiological data. A multiedge bandpass filter enables parallel fluorescence spectroscopy, allowing simultaneous detection and analysis of both exogenous and endogenous fluorophores. Coupled with multivariate regression analysis, we demonstrate the accurate quantification of fluorophore concentrations, facilitating comprehensive assessment of cardiac biochemical and functional dynamics. To mitigate geometric variability and motion artifacts, we developed a robust ratiometric approach using paired fluorescence agents. We demonstrate the system’s capability by employing the fluorescent lipophilic cation tetramethylrhodamine ethyl ester (TMRE) as a noninvasive biomarker for mitochondrial membrane potential, extracting physiologically relevant metrics. This platform enables sensitive assessment of cardiac function with established fluorescent probes and holds promising potential as a versatile tool for investigating the dynamics of novel fluorophores.

## Graphical abstract

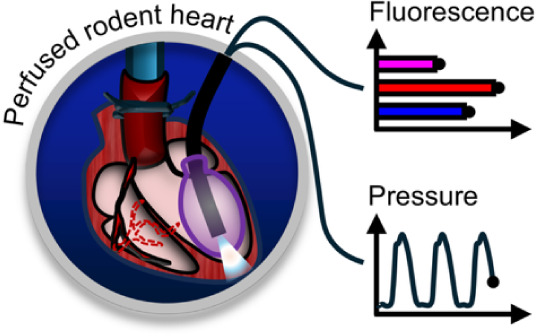

## Introduction

Cardiovascular diseases such as ischemia-reperfusion injury, myocardial infarction, heart failure, arrhythmias, and cardiomyopathies remain leading causes of morbidity and mortality worldwide, necessitating reliable experimental models for mechanistic and translational research. One of the most widely used tools in cardiovascular research is the Langendorff isolated perfused heart preparation,^1^ which enables investigation of cardiac physiology, electrophysiology, and biochemistry in an intact, beating heart. This model offers greater physiological relevance than cell culture systems while providing a level of experimental control and accessibility that is often unattainable in *in vivo* studies. Isolated heart perfusion systems are highly adaptable and well-suited for integration with a variety of biophysical, analytical, and imaging techniques, including Nuclear Magnetic Resonance (NMR) spectroscopy,^2,3^ nuclear molecular imaging approaches such as Positron Emission Tomography (PET) or Single Photon Emission Computed Tomography (SPECT),^4,5^ as well as optical/fluorescence imaging.

In the context of cardiovascular research, fluorescence imaging applied to isolated perfused hearts has provided valuable insights into biochemical processes under both physiological and pathological conditions, complementing findings from *in vitro* cell cultures and other *ex vivo* tissue studies. High-resolution microscopic techniques, such as confocal and two-photon microscopy, provide sufficient spatial resolution to visualize detailed myocyte morphology and even individual mitochondria.^6^ At the macroscopic level, widefield fluorescence imaging of Langendorff-isolated perfused hearts has transformed cardiac electrophysiology research by enabling visualization of electrical activity across the intact heart surface.^7,8^ Despite these advances, such imaging approaches predominantly rely on bulky, stationary benchtop systems, which are capable of interrogating only the epicardium and offer limited opportunities for multimodal imaging with complementary technologies such as NMR, PET, and SPECT.

Fiber-optic probes offer the capability to be anatomically positioned within the ventricular cavity, enabling the acquisition of localized information from the endocardium. This flexibility not only permits interrogation of otherwise inaccessible internal surfaces but also facilitates compatibility with NMR, PET, and SPECT. Such multimodal compatibility enables simultaneous acquisition of structural, functional, and biochemical data within a unified experimental framework, positioning fiber-optic fluorimetry as a powerful tool for comprehensive, high-resolution cardiac phenotyping in both basic and translational research.^9^ Fiber-optic lifetime and steady-state fluorescence technologies have been developed for monitoring autofluorescence (NADH, FAD),^10^ [Ca^2+^],^11^ and pO_2_,^12^ but most multiparametric implementations rely on interleaved imaging either by switching light sources periodically^8,10,13^ or by rotating a filter wheel,^14,15^ which limits their temporal resolution and capacity to monitor rapid biochemical processes. A persistent challenge in cardiac fluorescence imaging remains the correction of motion artifacts. Various hardware-based strategies have been employed to address this issue, including chemically uncoupling cardiac contraction,^6^ mechanical stabilization of tissue motion,^16^ synchronized detection with the cardiac cycle,^17^ temporal averaging to smooth transient fluctuations,^10^ spatial averaging to reduce local variability,^11,18^ and ratiometric techniques to normalize against geometric and intensity fluctuations.^19^ Mechanical or chemical stabilization limits the physiological relevance of the resultant measurements. Synchronized detection and temporal averaging become unreliable under arrhythmic conditions, and spatial averaging, such as with integrating spheres, is difficult to miniaturize. Ratiometric measurements typically require simultaneous recording of a nonspecific fluorophore, used as a geometry reference, and a molecular-specific fluorophore of interest. However, this approach can be limited by differences in their respective tissue loading properties and pharmacokinetics.

Here, we present a multiexcitation fiber-optic spectroscopic platform for monitoring multiple fluorophores in perfused hearts. We developed a single fiber-optic probe for both illumination and collection that can be inserted into the left ventricular (LV) lumen via a fluid-filled catheter. The distal tip of the probe integrates an inflatable balloon, enabling the simultaneous measurement of LV pressure, heart rate, and endocardial fluorescence through concurrent excitation and collection. Fluorescence data were analyzed using a computational model based on multiple linear regression (MLR) of a reference library of fluorophores for abundance estimation. This spectroscopic approach enables the removal of autofluorescence background, correction of motion artifacts, and multiparametric sensing. We demonstrate the system using the lipophilic cationic fluorophore tetramethylrhodamine ethyl ester (TMRE) to longitudinally monitor mitochondrial membrane potential in the beating isolated perfused rat heart.

## Experimental Section

### Multiexcitation Fiber-Optic Fluorescence Spectroscopy

A schematic of the custom-designed system for multiexcitation spectroscopic monitoring of Langendorff-perfused rodent hearts is shown in Figure 1a. The optical excitation module employs three high-power LEDs (M470F3, MINTF4, M625F2; Thorlabs, Inc.) operating at center wavelengths of 470, 543, and 625 nm, respectively. Each LED output is individually collimated and passed through dedicated band-pass filters (FF01-474/27-25, FF01-543/22-25, FF01-546/SP-25, and FF01-650/13-25; Semrock, Inc.) to ensure spectral purity and minimize cross-excitation. The spectrally filtered beams are sequentially combined using a cascade of dichroic mirrors (MGP01-350-700-25 × 36, FF505-SDi01-25 × 36, and FF625-SDi01-25 × 36; Semrock, Inc.), forming a single, multiplexed excitation path. This combined excitation beam is directed onto a triple-edge dichroic beam splitter (Di01-R488/543/635–25 × 36; Semrock, Inc.), which reflects the excitation wavelengths while transmitting the longer-wavelength fluorescence emission. The reflected excitation light is then coupled to the optical fiber of the balloon probe, enabling delivery of excitation light directly to the endocardial surface of the isolated heart. Fluorescence emitted from the tissue is collected by the same fiber and passes back through the triple-edge dichroic filter before being directed into a fiber-coupled spectrometer (QE Pro; Ocean Insight) for spectral analysis. To suppress residual excitation light and reduce background noise, a multinotch emission filter (FF01-446/510/581/703-25; Semrock, Inc.) is placed in front of the spectrometer. Additionally, a set of linear polarizers (LPVISE100-A; Thorlabs, Inc.) is employed to further attenuate specular reflections and enhance the signal-to-noise ratio. This optical configuration enables high-sensitivity, real-time acquisition of spectrally resolved fluorescence signals from multiple fluorophores, facilitating simultaneous biochemical and functional assessment of cardiac tissue.

**Figure 1:**
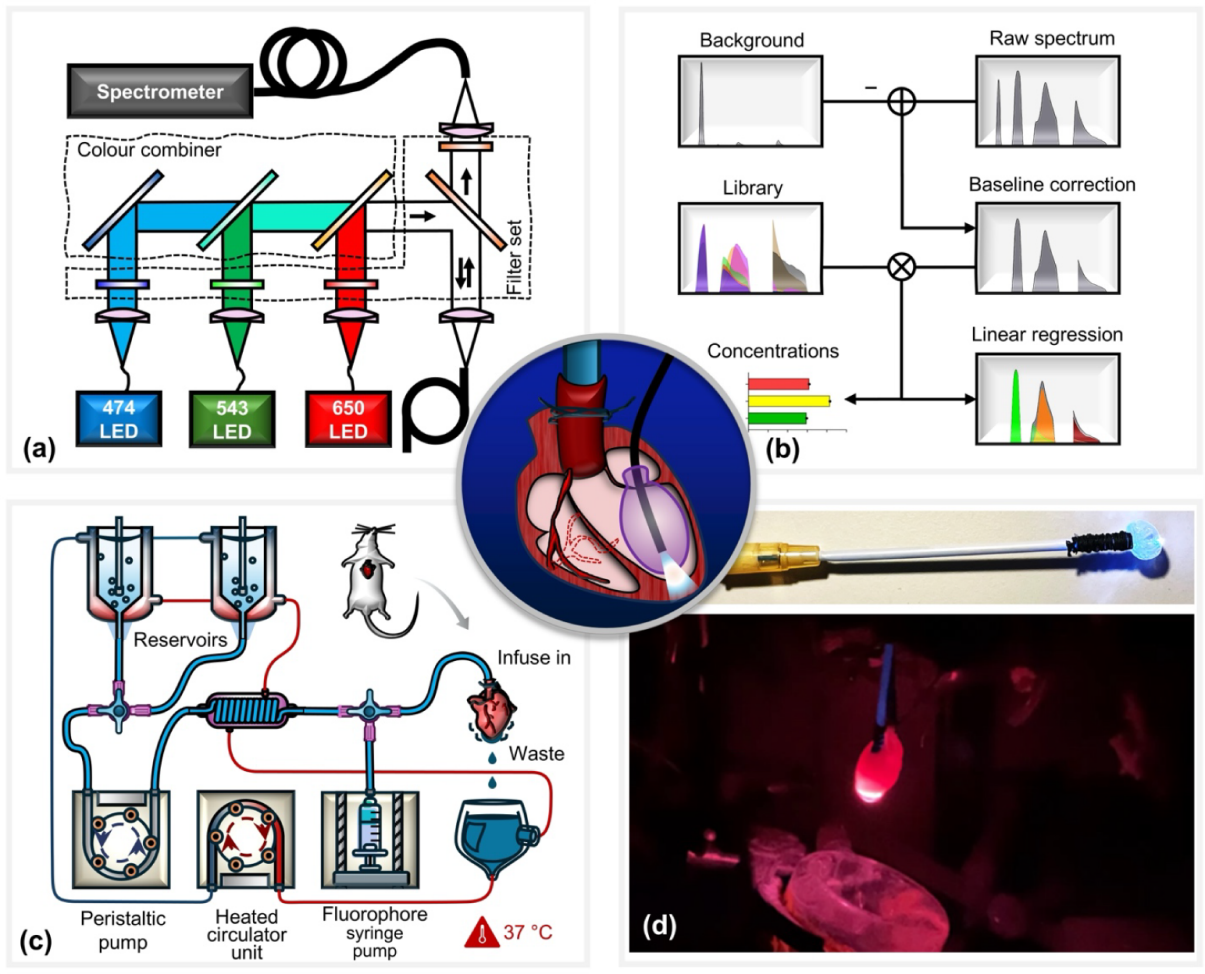
(a) Schematic of the multiexcitation fiber-optic platform. Light from three spectrally filtered LEDs is optically combined and coupled into a single-core multimode fiber for simultaneous three-color excitation. The same fiber is used for both excitation and collection of endocardial fluorescence. A multiedge bandpass filter enables parallel spectral acquisition without the need for source switching. (b) Data processing workflow for spectral unmixing and estimation of relative fluorophore concentrations from acquired spectra using multivariate regression. (c) Langendorff heart perfusion system. Two switchable reservoirs supply perfusate equilibrated with either 95% O_2_/5% CO_2_ or 95% N_2_/5% CO_2_. A peristaltic pump maintains a constant perfusion rate (14 mL/min); a water bath heats the perfusate to physiological temperature, and a syringe pump delivers fluorophores for controlled loading. (d) Photographs of the fiber-optic balloon probe. The water-filled balloon is inserted into the left ventricle (LV) via the left atrium and inflated in situ for intraventricular pressure measurement. The embedded optical fiber enables concurrent epi-fluorescence detection from the endocardium.

### Cardiac Fiber-Optic Balloon Probe

The balloon probe was constructed from a multimode optical fiber (FG550LEC, 0.22 NA, Thorlabs, Inc.), featuring a flat-cleaved, bare fiber tip enclosed within a water-filled elastomeric balloon. The balloon was designed to expand to a diameter of approximately 3–5 mm, allowing insertion into the left ventricular (LV) lumen through a small incision made in the left atrium. Once positioned, the balloon is inflated using a 1 mL syringe connected via a three-way luer-lock valve to both a pressure transducer and the balloon through water-filled PVC tubing, enabling simultaneous intraventricular pressure measurement. The single optical fiber housed inside the balloon serves dual roles for both fluorescence excitation and emission collection. By gently retracting the probe, the fiber tip can be positioned approximately 1 mm from the endocardial surface, allowing localized, stable, and minimally invasive optical interrogation of the internal ventricular wall under near-physiological conditions.

### Computational Fluorescence Deconvolution

We developed a data processing pipeline that estimates the abundances of individual fluorophores (Figure 1b). We constructed a linear model $y_{1\times N}=c_{1\times M}\cdot X_{M\times N}$ , where **y** represents the measured fluorescence spectrum, **X** is the library of reference spectra, *N* is the number of wavelengths, and *M* is the number of fluorophores included in the model. The concentration coefficients **c** were determined by solving this equation using least-squares regression. To obtain the measured fluorescence spectrum **y**, the raw fluorescence data were preprocessed using background subtraction, followed by a time-averaging filter. The background spectrum, accounting for the system’s internal background and autofluorescence, was obtained by averaging autofluorescence spectra before any exogenous fluorophore infusion. To mitigate motion artifacts induced by heartbeats, a one-second time-averaging filter was applied to the fluorescence data. The library spectra of **X** consisting of emission spectra of individual fluorophores were collected in perfused hearts.

### Langendorff Perfusion Experiment

All experimental procedures were approved by King’s College London’s local Animal Care and Ethics Committee and carried out in accordance with Home Office regulations as detailed in the Guidance on the Operation of Animals (Scientific Procedures) Act 1986. Rats were coadministered sodium pentobarbital (200 mg kg^–1^) and sodium heparin (200 IU kg^–1^) by intraperitoneal injection. Hearts were excised and immediately arrested in ice-cold Krebs–Henseleit buffer (KHB) consisting of (mmol L^–1^): NaCl, 118; KCl, 5.9; MgSO_4_, 1.16; NaHCO_3_, 25; NaEDTA, 0.48; glucose, 11.1; and CaCl_2_, 2.2. The aorta was cannulated and secured using 3–0 suture (Ethicon), and the pulmonary artery was incised to drain coronary effluent and perfused with KHB gassed with 95%O_2_/5%CO_2_ at 37 °C, with perfusion maintained at a constant rate of 14 mL/min, as we have described previously.^20^ The schematic of the perfusion system is shown in Figure 1c. The fiber-balloon catheter (Figure 1d) was inserted gently into the left ventricle via the left atrium and inflated with water using a spindle syringe, as shown in the center panel of Figure 1. The perfusion pressure and the left ventricle pressure (LVP) were monitored at 400 Hz using two pressure transducers connected to a PowerLab data acquisition system (ADInstruments Ltd.), from which the left ventricular end-diastolic pressure (LVEDP), heart rate (HR), and the left-ventricular-developed pressure (LVDP) were derived. Once each heart preparation had established its inclusion criteria (successful cannulation and balloon insertion within 5 min, stable sinus rhythm developing >100 mmHg LVDP with a LVEDP of 6 ± 2 mmHg and a coronary perfusion pressure of 70–85 mmHg), optical recording was initiated at 0.5–100 Hz, controllable by the integration time. Freshly prepared fluorophores diluted in KHB were injected by a syringe driver via a side arm at 1.4 mL/min (i.e., 10%), with the coronary perfusion rate correspondingly decreased by 1.4 mL/min (to 90%). Interventions such as hypoxia were achieved by switching to the reservoir gassed with 95% N_2_/5% CO_2_, while pharmacological interventions such as carbonyl cyanide *m*-chlorophenylhydrazone (CCCP) infusion were achieved by parallel side arm infusion or addition to the perfusate reservoir as appropriate. All fluorescence spectra and hemodynamic data were analyzed and monitored by MATLAB (The MathWorks, Inc.) and LabChart (ADInstruments Ltd.), respectively, both of which gave instant data visualization.

### Fluorophore Synthesis

Tokyo Green (TG) and 1,3-Dichloro-7-hydroxy-9,9-dimethylacridin-2­(9H)-one (DDAO) were synthesized in-house as described previously.^21^ Detailed synthetic protocols and characterization data can be found in the Supporting Information (Materials and Methods).

### System Background Characterization

The excitation light source is approximately one order of magnitude more intense than the fluorescence emission, meaning that even a small fraction of excitation photons reaching the detector can generate significant background signals and potentially cause detector saturation. To address this, we first characterized the system background, consisting of two components: (i) a constant contribution from internal system reflections independent of the tissue and (ii) a dynamic contribution from tissue-specific reflections arising from variations in optical properties. While the constant background component can be removed by simple subtraction, the dynamic component is more difficult to eliminate because it varies with changes in cellular metabolism. We measured the reflection profile from the fiber tip, where the internal background primarily originated from back-reflection at the fiber’s proximal end and autofluorescence from the fiber’s plastic coating or jacket (Figure 2a). This analysis revealed three emission bands: 495–525 nm, 548–621 nm, and 670–749 nm, with negligible dynamic background, as distal-end reflections remained below 1600 analog-to-digital units (ADUs), corresponding to less than 0.8% of the detector’s full dynamic range.

**Figure 2:**
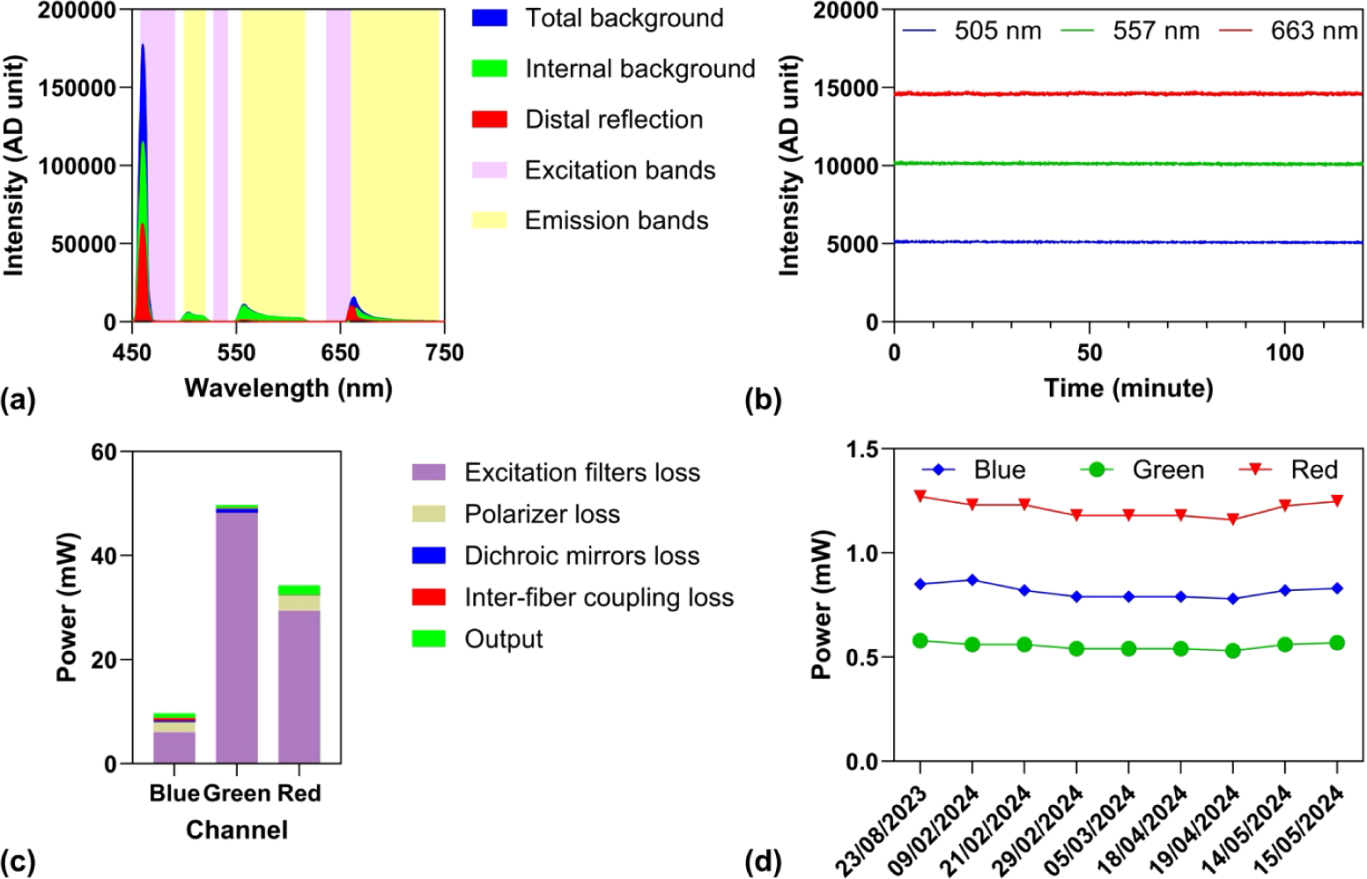
(a) Spectral analysis of the system background. The background signal comprises a constant internal component and a distal reflection component (green area) dependent on tissue optical properties. For fluorescence measurements, spectral regions with minimal reflection background were selected: 495–525 nm, 548–621 nm, and 670–749 nm. (b) Stability of excitation sources over a 2-h continuous recording shows minimal intensity noise, confirming low intrinsic fluctuations and shot-noise-limited performance. (c) Power budget analysis indicates that the primary losses occur in the excitation filters, while fiber-to-fiber coupling losses remain low. (d) Daily measurements reveal minor long-term drifts in LED output, suggesting the need for periodic normalization or correction for high-precision experiments.

### System Stability and Efficiency

Accurate measurement of fluorophore dynamics with minimal interference from source fluctuations and detector noise requires a highly stable excitation source and optimized collection efficiency. To assess the stability of the light source, we continuously recorded the system background with the spectrometer over a 2 h period following an LED warm-up phase. The spectrometer was configured with a 1 s integration time, consistent with the integration time used in subsequent perfusion experiments. Temporal intensity fluctuations of 505, 557, and 663 nm LEDs are shown in Figure 2b. The root-mean-square (RMS) noise levels were 29.7, 40.3, and 46.5 ADUs at the respective wavelengths. These values are consistent with the shot noise behavior expected for a typical photodetector, indicating that the LEDs exhibit low intrinsic noise and enable shot-noise-limited detection. Intensity fluctuations recorded at a sample rate of 100 Hz show similar noise levels (Figure S1). To evaluate light transmission efficiency, we measured optical power at multiple points along the excitation path using a calibrated power meter: directly at the LEDs, after the excitation filters, following the dichroic mirrors, and at the distal tip of the fiber. Using these measurements along with the nominal transmittance of the polarizer, we quantified power losses and calculated the efficiency of the excitation filters, dichroic mirrors, polarizer, and fiber-to-fiber coupling. The resulting optical power budget, shown in Figure 2c, indicates that the majority of the power loss occurs at the excitation filters. The measured excitation powers for the blue, green, and red channels were 0.91, 0.62, and 1.83 mW, respectively, resulting in a total irradiance of 0.43 W/cm^2^ at a distance of 1 mm from the fiber tip. This level of irradiance is unlikely to cause significant photobleaching of fluorophores or thermal damage to the tissue. The estimated fiber-to-fiber coupling efficiencies were 65%, 79%, and 97% for the respective channels, as summarized in Table 1.

**Table 1:** Light Efficiency in the Illumination Path

		Blue channel	Green channel	Red channel
	Power in (mW)	9.7	49.7	34.3
Transmittance (%)	Excitation filters	38	3.0	14
	Polarizer	42^a^	n/a	40^a^
	Dichroic mirrors	91	53	97
	Interfiber	65	79	97
	Power out (mW)	0.91	0.62	1.83

a Extracted from the nominal data from the supplier.

In our experiments, we observed two main factors that can influence reproducibility: (1) drift in the excitation light sources and (2) variations in the fiber coupling system. Specifically, the output power of LEDs can fluctuate depending on thermal dissipation conditions, as they operate in the open-loop mode. In addition, the coupling efficiency of the optical system may decrease due to angular deviations of dichroic mirrors, which can result from mechanical vibrations or the gradual release of internal stress. These effects typically occur over longer time scales (>2–24 h), while in the short term, noise levels remain low (Figure 2b). To ensure long-term reproducibility, we calibrated the output power from the fiber probe for each LED prior to every perfusion experiment and applied a software correction. This procedure also compensates for manufacturing variability in the fiber-based sensor.

### Spectral Library Calibration

Accurate estimation of fluorophore abundance relies on a well-calibrated reference library. Building such a library, however, is challenging because fluorescence emission is strongly affected by tissue absorption and scattering. In addition, tissue-specific molecular interactions can further alter the signal. To account for these effects, the reference library was generated from measurements in isolated perfused hearts rather than from pure fluorophore solutions. The calibration of exogenous fluorophores TMRE, TG, and DDAO was conducted by directly loading the exogenous fluorophores into the tissue. For endogenous fluorophores, such as FAD, we perfused hypoxic KHB at a constant flow rate. Figure 3a,b compares spectral libraries from pure fluorophore solutions and from isolated hearts, respectively, illustrating how tissue absorption modifies emission spectra. Assuming for a first approximation that the diluted pure solutions are transparent, the effect of tissue absorption can be quantified by subtracting the emission spectra of pure solutions from those of the isolated hearts, as shown in Figure 3c.

**Figure 3:**
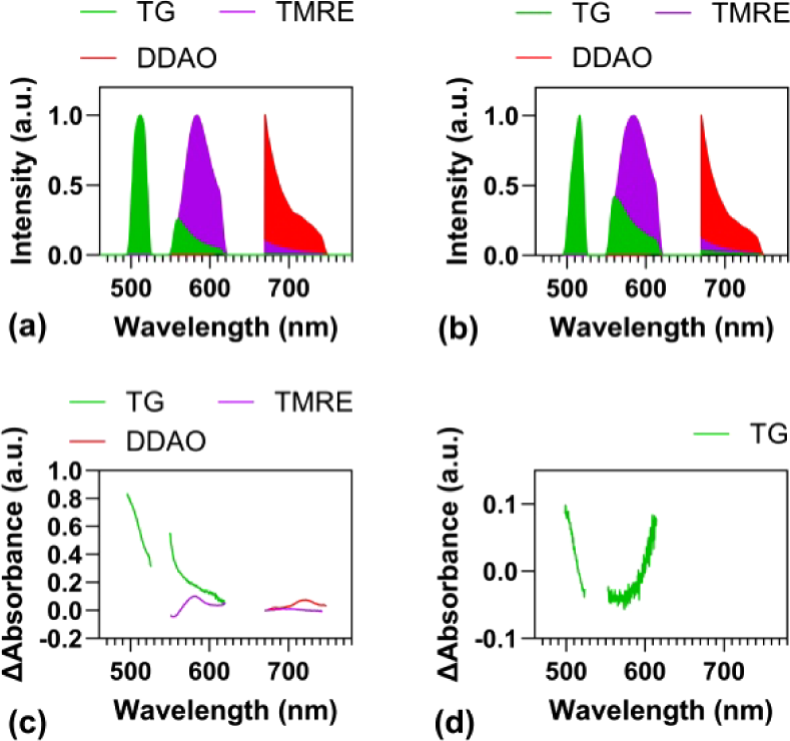
(a) Reference fluorescence spectra of individual fluorophores measured in pure solution. (b) Corresponding spectra measured in Langendorff-perfused hearts under normoxic conditions. (c) Differential spectra between heart and solution measurements, highlighting spectral distortion due to tissue scattering and absorption. (d) Differential spectra between normoxic and hypoxic hearts, shown in optical density units, revealing changes associated with the oxygenation state.

There are still limitations in this approach since the absorbance spectrum varies depending on the fluorophore, which may result from both tissue absorption and fluorescence reabsorption. Additionally, baseline tissue absorption is likely influenced by the heart’s metabolic state, particularly during hypoxia.^18^ Since one potential application of this system is to screen and test hypoxia-sensing fluorophores, we therefore developed a spectral library for tissue hypoxia. The absorbance difference for TG under hypoxia versus normoxia is shown in Figure 3d, with a maximum difference of 0.1 optical density.

## Results and Discussion

### Characterization of Fluorophore Permeability, Stability, and Equilibration Kinetics in Perfused Hearts

We first conducted perfusion experiments to test the cell permeability, stability, and equilibrium rate of DDAO and TG fluorophores in normal hearts. Fluorescence intensities exhibited an initial exponential increase during fluorophore loading (Figure 4a–d). Equilibrium, where influx and efflux rates balance, was reached after 40 min infusion for 50 nM DDAO (Figure 4a) or after 10 min infusion for 50 nM TG (Figure 4c). We modeled the equilibration using the equation $I_{F}/a=1-e^{-\left(t-t_{0}\right)/\tau }$ , derived from a two-compartmental model, where *I*_F_ is the fluorescence intensity, *a* is the normalization coefficient, *t*_0_ is the lag time, and τ is the time constant. A larger time constant indicates longer equilibration. Curve fitting yielded time constants of 17.8 min for DDAO and 2.59 min for TG at an infusion concentration of 50 nM. Higher infusion concentrations (Figure 4b,d) resulted in significantly shorter equilibration times compared to lower concentrations (Figure 4a,c). These results show that both DDAO and TG are permeable and stable in cardiac tissue, while DDAO takes a longer time than TG to reach equilibrium.

**Figure 4:**
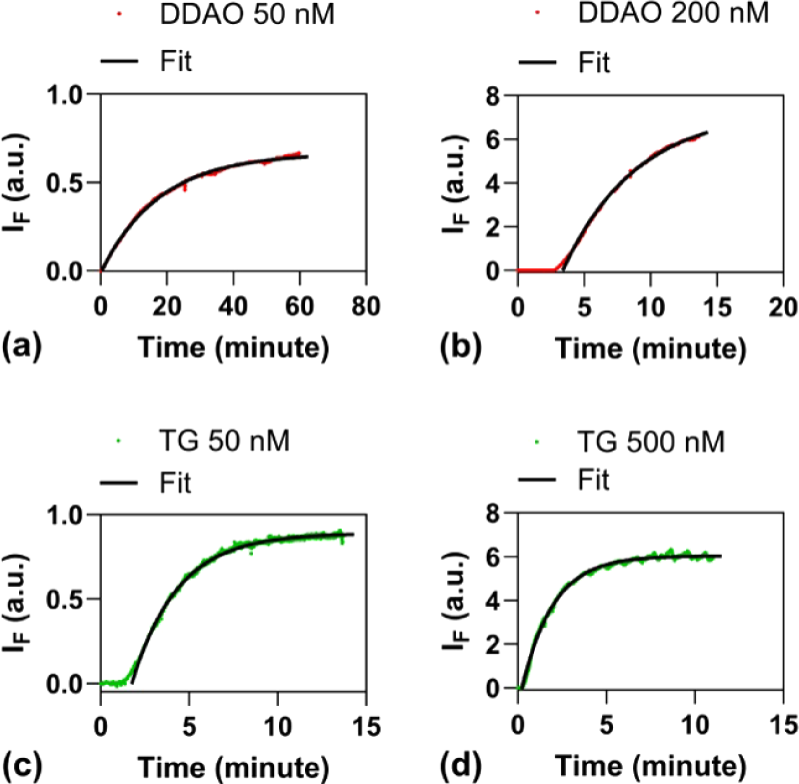
DDAO 50 nM (a), DDAO 200 nM (b), TG 50 nM (c), and TG 500 nM (d) in perfused hearts. *I*_F_ is the fluorescence intensity. The curves were fitted by the equation $I_{F}/a=1-e^{-\left(t-t_{0}\right)/\tau }$ , derived from a two-compartmental model, where *a* is the normalization coefficient, *t*_0_ is the lag time, and τ is the time constant. The time constants are 17.8 min for DDAO 50 nM, 5.57 min for DDAO 200 nM, 2.59 min for TG 50 nM, and 1.77 min for TG 500 nM.

### Validation of the System with TMRE: Monitoring Mitochondrial Membrane Potential and Cardiac Function

We then validated the system using a well-characterized and widely studied fluorophore. TMRE is a membrane-permeable, fast-equilibrating, lipophilic cationic fluorophore which accumulates within mitochondria according to their membrane potential (Δψ_*m*_) and is widely used in cell culture as an indicator of both mitochondrial and cellular viability.^22^ At low concentrations (nonquenching), the TMRE signal is linearly dependent on fluorophore concentration. However, at high concentrations (quenching), TMRE molecules undergo π–π stacking, leading to concentration-dependent quenching and nonlinear signal saturation. The mitochondrial component of TMRE signals in experiments of this sort is usually determined by depolarizing the mitochondria with ionophores like CCCP (carbonyl cyanide *m*-chlorophenyl hydrazone), leading to TMRE washout and a loss of signal. At low concentrations (nonquenching), this washout is typically exponential, but at higher concentrations, an initial washout of TMRE would lead to initial unquenching and a “fluorescence burst” prior to the exponential loss of signal as intramitochondrial TMRE washes out. With this insight, we conducted comparative assessments of mitochondrial loading and CCCP-induced TMRE washout to characterize and validate the parallel fluorescence and contractile readouts of our system with TMRE infused at low concentration (2 nM, nonquenching), intermediate concentration (20 nM), and quenching mode (high concentrations (100 nM, quenching).

When infused at 2 nM, cardiac TMRE fluorescence increased linearly during the loading phase and remained stable throughout the washout period (Figure 5a). This indicates the stable retention of the fluorophore within polarized mitochondria. Cardiac contractility remained relatively unaffected throughout the experiment (Figure 5a). Upon exposure to the mitochondrial uncoupler CCCP, TMRE fluorescence rapidly declined due to fluorophore washout, coinciding with a sharp reduction in left ventricular developed pressure and a progressive rise in left ventricular end-diastolic pressure (Figure 5d). When infused at 20 nM, TMRE fluorescence increased during loading more rapidly but slowly washed out in the ensuing washout period, coupled with a progressive loss of left ventricular developed pressure and increased end diastolic pressure, suggestive of mitochondrial toxicity of the higher concentration of fluorophore (Figure 5b). When hearts were exposed to 600 nM CCCP, the fluorescence initially increased during the early washout phase, followed by a progressive decline, suggesting an initial unquenching effect prior to fluorophore washout (Figure 5e). When infused at 100 nM, TMRE fluorescence again loaded linearly and then slowly washed out, with a corresponding progressive loss of left ventricular developed pressure (Figure 5c). However, CCCP-induced mitochondrial depolarization resulted in a significant increase in TMRE fluorescence, as the fluorophore became unquenched as its intramitochondrial concentration fell (Figure 5f). Interestingly, the tissue autofluorescence signal, tentatively attributed to FAD, showed marked fluctuations in control hearts (Figure 5a,b) and consistently decreased following CCCP infusion (Figure 5d,e). In 2 of 19 experiments, an additional autofluorescence component was observed (red curve in Figure 5b), with an emission peak near 680 nm (Figure S2), although its origin remains unknown. These autofluorescence signals were effectively separated using our MLR analysis, highlighting the method’s ability to resolve weak signals within a variable and spectrally overlapping autofluorescence background. Overall, these results demonstrate that our miniaturized balloon probe reliably captured both TMRE fluorescence and intraventricular pressure from the endocardium in normal and CCCP-treated hearts. The observed TMRE dynamics during loading and in response to CCCP are consistent with previous reports.^22^ The high sensitivity of the system enables monitoring of mitochondrial membrane potential using low TMRE concentrations, thereby minimizing cellular toxicity.

**Figure 5:**
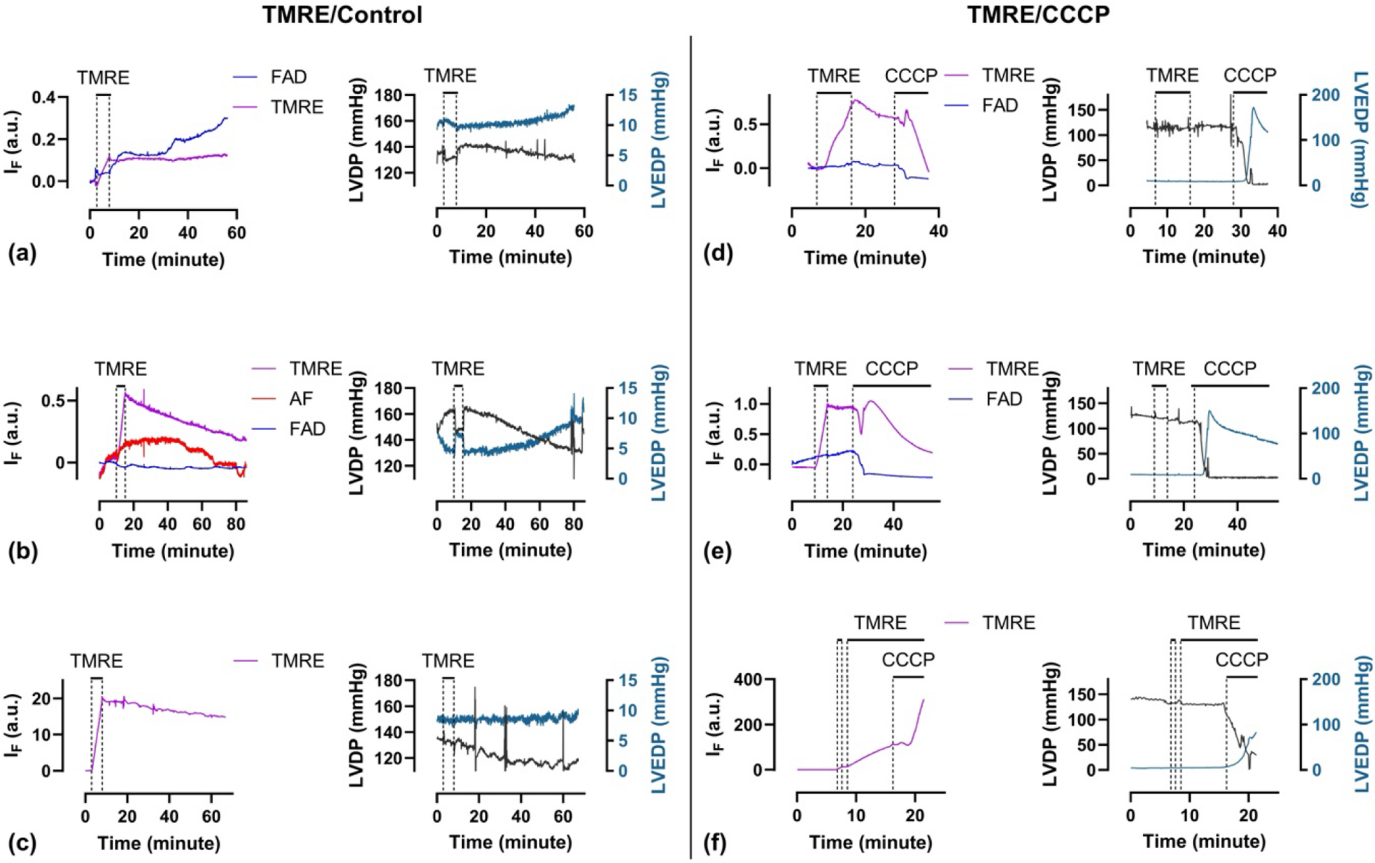
TMRE/control group with TMRE concentrations of 2 nM (a), 20 nM (b), and 100 nM (c). The TMRE/CCCP group with TMRE concentrations of 2 nM (d), 20 nM (e), and 100 nM (f). *I*_F_, fluorescence intensity; LVDP, left-ventricular-developed pressure; LVEDP, left ventricular end-diastolic pressure; and AF, unknown autofluorescence.

### Ratiometric Correction of Geometric Artifacts Using a Reference Fluorophore

Motion artifacts are generally minimal in stable hearts with a normal rhythm. However, we detected several signal spikes with distinct temporal characteristics, likely resulting from arrhythmic events. While these artifacts did not significantly impact the experiments described here, they could pose challenges for the absolute quantification of fluorophores, particularly under interventions such as hypoxia, which may induce baseline shifts. Therefore, the ability to correct geometric changes within our system was highly desirable. Ratiometric dyes offer one potential solution; however, their absorption and emission spectra often change nonlinearly upon binding to target ions or molecules, complicating calibration for MLR analysis. To address this, we investigated the system’s ability to unmix two simultaneously infused fluorophores, using one as an inert “reference” fluorophore to correct for changes in geometry, contractility, and perfusion. Ideally, such reference fluorophores should be biologically insensitive to the target of interest, yet responsive to potential experimental confounders that influence fluorophore pharmacokinetics more broadly.

DDAO is a cell-permeable, photostable fluorophore with an emission peak at 658 nm, well separated from the fluorescence of both TMRE and FAD. Because of this spectral separation, DDAO was selected as a reference fluorophore for the ratiometric measurement of TMRE. Using DDAO as a reference, we aimed to correct geometric factors that affect fluorescence intensity. These include differences in probe-to-tissue geometry between hearts as well as dynamic changes within the same heart due to contraction. To evaluate the robustness of this approach under extreme conditions, we deliberately altered the probe position. In one case, the fiber was placed close to the tissue (Figure 6a), and in another, it was positioned further away (Figure 6b). Before normalization, the raw TMRE signal in Figure 6a is more than twice as strong as that in Figure 6b, even though both experiments used the same TMRE concentration and loading duration. In addition, we observed fluctuations where the TMRE and DDAO signals rose or fell in parallel, suggesting that these were likely due to motion or perfusion artifacts rather than biological signals. After normalizing the TMRE by the DDAO reference signal, the ratiometric results (Figure 6c) provided a more accurate representation of TMRE behavior. Notably, both experiments showed TMRE signals converging to the same level by the end of the loading period, indicating effective correction of geometry-related variability. Moreover, the sharp signal increase in the TMRE/CCCP group was no longer preceded by an apparent drop, as seen in the raw signal (Figures 5d–f and 6b). This suggests that the initial valleys were likely artifacts that were successfully removed by the normalization process.

**Figure 6:**
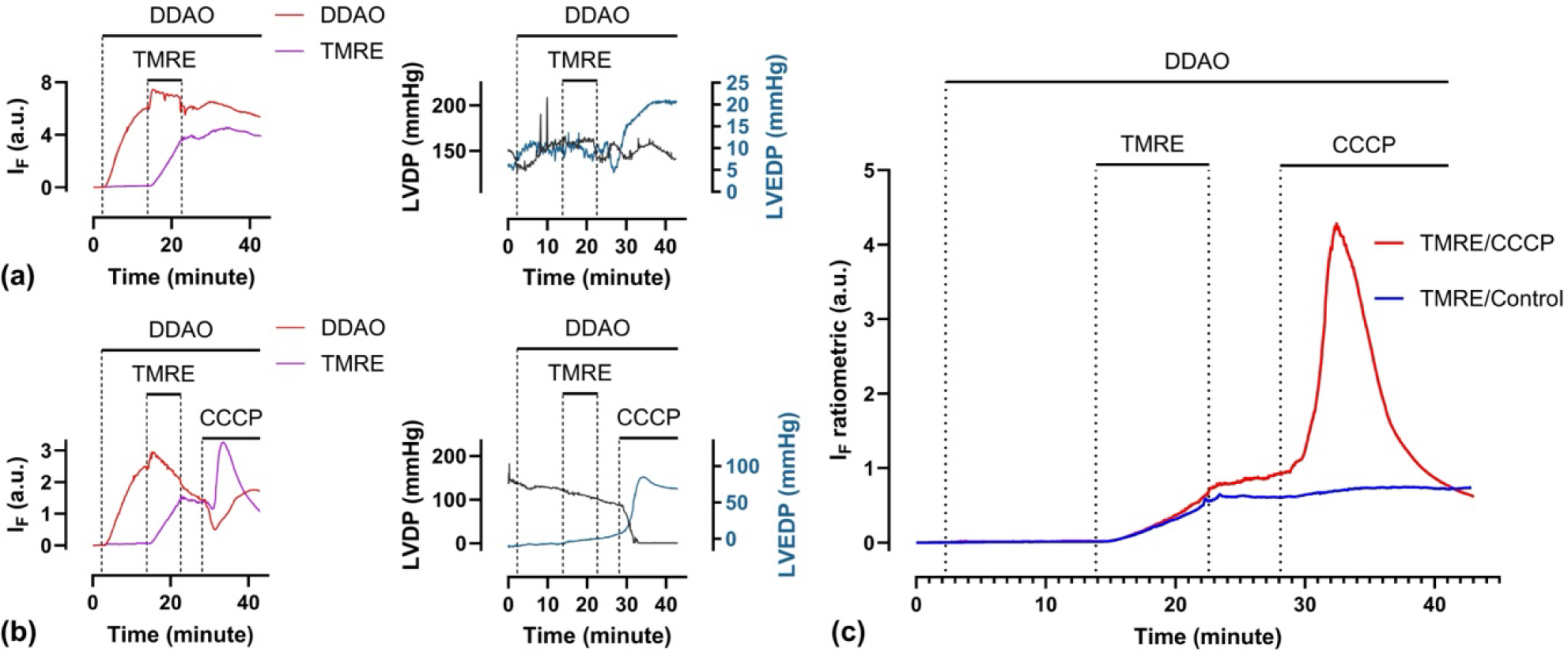
Hearts were loaded with 200 nM DDAO as the reference fluorophore and 20 nM TMRE without (a) and with (b) 300 nM CCCP. (c) Normalized TMRE fluorescence to that of DDAO. *I*_F_, fluorescence intensity; LVDP, left-ventricular-developed pressure; LVEDP, left ventricular end-diastolic pressure.

Good spectral fits from one perfused heart can be achieved using a spectral library calibrated from other hearts, underscoring the robustness of computational spectral deconvolution despite variations in tissue scattering and absorption (Supplementary video). The main challenge in fluorescence tissue analysis still arises from scattering and absorption in tissues. Unlike diluted fluorophore solutions, where these effects are negligible, tissue significantly alters both excitation and emission light. Several strategies may mitigate these effects. For example, diffuse reflectance spectroscopy can be combined with epi-fluorescence to improve interpretability, although this requires additional hardware.^23^ Restricting analysis to the near-infrared range is also effective but limited to red or infrared fluorophores.^11^ A distinctive feature of our probe design is the use of a single fiber for both excitation and collection. Unlike fiber bundles that separate these functions, our zero-spatial-offset configuration samples only the most superficial tissue volume and is therefore less sensitive to diffusely scattered photons.^24^

## Conclusions

We have developed a multiexcitation fiber-optic spectroscopy system for real-time monitoring of dynamic fluorescence signals in isolated Langendorff-perfused rat hearts. By using multiple excitation wavelengths and a high-efficiency spectroscopic setup, the system enables parallel tracking of multiple fluorophores with nanomolar sensitivity at a temporal resolution of 1 Hz. The system incorporates a fiber-optic balloon catheter that allows the simultaneous acquisition of intraventricular pressure and endocardial fluorescence, enabling direct correlation between cardiac biochemical signals and mechanical function. We implemented a multivariate spectral deconvolution approach for resolving individual fluorophore signals from mixed emission spectra. This analysis facilitates background autofluorescence removal, detection of unknown spectral components, and ratiometric correction for geometry-related artifacts and dynamic changes in tissue perfusion. The system is readily compatible with integration into multimodal imaging platforms, such as optical-PET, SPECT, and NMR. In addition, its catheter-based design offers adaptability for minimally invasive or intravital imaging in other organ systems.

## Supplementary Material





## Data Availability

Data underlying the results presented in this paper are not publicly available at this time but may be obtained from the authors upon request.

## Notes

Residuals of spectral fit to one perfused heart using multiple linear regression (MP4)
Synthesis of DDAO and Tokyo Green; stability of excitation sources recorded at 100 Hz; and the spectrum of unknown autofluorescence (PDF)

## References

[1] Bell R. M., Mocanu M. M., Yellon D. M. (2011). Retrograde Heart Perfusion: The Langendorff
Technique of Isolated Heart Perfusion. J. Mol.
Cell. Cardiol..

[2] Eykyn T. R., Aksentijević D., Aughton K. L., Southworth R., Fuller W., Shattock M. J. (2015). Multiple Quantum Filtered 23Na NMR
in the Langendorff Perfused Mouse Heart: Ratio of Triple/Double Quantum
Filtered Signals Correlates with [Na]­i. J. Mol.
Cell. Cardiol..

[3] Weiss K., Mariotti E., Hill D. K., Orton M. R., Dunn J. T., Medina R. A., Southworth R., Kozerke S., Eykyn T. R. (2012). Developing
Hyperpolarized 13C Spectroscopy and Imaging for Metabolic Studies
in the Isolated Perfused Rat Heart. Appl. Magn.
Reson..

[4] Mariotti E., Veronese M., Dunn J. T., Medina R. A., Blower P. J., Southworth R., Eykyn T. R. (2013). Assessing Radiotracer
Kinetics in
the Langendorff Perfused Heart. EJNMMI Res..

[5] Southworth R., Dearling J. L., Medina R. A., Flynn A. A., Pedley B. R., Garlick P. B. (2002). Dissociation of
Glucose Tracer Uptake and Glucose Transporter
Distribution in the Regionally Ischaemic Isolated Rat Heart: Application
of a New Autoradiographic Technique. Eur. J.
Nucl. Med. Mol. Imaging.

[6] Matsumoto-Ida M., Akao M., Takeda T., Kato M., Kita T. (2006). Real-Time
2-Photon Imaging of Mitochondrial Function in Perfused Rat Hearts
Subjected to Ischemia/Reperfusion. Circulation.

[7] Wang L., Ripplinger C. M. (2019). Putting the Pieces Together Using in Vivo Optical Mapping. Cardiovasc. Res..

[8] Caldwell J. L., Lee I.-J., Ngo L., Wang L., Bahriz S., Xu B., Bers D. M., Navedo M. F., Bossuyt J., Xiang Y. K., Ripplinger C. M. (2023). Whole-Heart
Multiparametric Optical Imaging Reveals
Sex-Dependent Heterogeneity in cAMP Signaling and Repolarization Kinetics. Sci. Adv..

[9] Weissleder R., Pittet M. J. (2008). Imaging in the Era
of Molecular Oncology. Nature.

[10] Lagarto J. L., Dyer B. T., Talbot C. B., Peters N. S., French P. M. W., Lyon A. R., Dunsby C. (2018). Characterization of NAD­(P)H and FAD
Autofluorescence Signatures in a Langendorff Isolated-Perfused Rat
Heart Model. Biomed. Opt. Express..

[11] Kosmach A., Roman B., Sun J., Femnou A., Zhang F., Liu C., Combs C. A., Balaban R. S., Murphy E. (2021). Monitoring Mitochondrial
Calcium and Metabolism in the Beating MCU-KO Heart. Cell Rep..

[12] Griffiths J. R., Robinson S. P. (1999). The OxyLite: A Fibre-Optic Oxygen
Sensor. Br. J. Radiol..

[13] Kappadan V., Telele S., Uzelac I., Fenton F., Parlitz U., Luther S., Christoph J. (2020). High-Resolution
Optical Measurement
of Cardiac Restitution, Contraction, and Fibrillation Dynamics in
Beating vs. Blebbistatin-Uncoupled Isolated Rabbit Hearts. Front. Physiol..

[14] Brachmanski M., Gebhard M. M., Nobiling R. (2004). Separation of Fluorescence Signals
from Ca2+ and NADH during Cardioplegic Arrest and Cardiac Ischemia. Cell Calcium.

[15] Staniszewski K., Audi S. H., Sepehr R., Jacobs E. R., Ranji M. (2013). Surface Fluorescence
Studies of Tissue Mitochondrial Redox State in Isolated Perfused Rat
Lungs. Ann. Biomed. Eng..

[16] Morishita Y., Tamura S., Mochizuki K., Harada Y., Takamatsu T., Hosoi H., Tanaka H. (2023). Generation
of Myocyte Agonal Ca2+
Waves and Contraction Bands in Perfused Rat Hearts Following Irreversible
Membrane Permeabilisation. Sci. Rep..

[17] Kuo C. W., Pratiwi F. W., Liu Y.-T., Chueh D.-Y., Chen P. (2022). Revealing
the Nanometric Structural Changes in Myocardial Infarction Models
by Time-Lapse Intravital Imaging. Front. Bioeng.
Biotechnol..

[18] Bauer T. M., Giles A. V., Sun J., Femnou A., Covian R., Murphy E., Balaban R. S. (2019). Perfused
Murine Heart Optical Transmission
Spectroscopy Using Optical Catheter and Integrating Sphere: Effects
of Ischemia/Reperfusion. Anal. Biochem..

[19] Lee P., Quintanilla J. G., Alfonso-Almazán J. M., Galán-Arriola C., Yan P., Sánchez-González J., Pérez-Castellano N., Pérez-Villacastín J., Ibañez B., Loew L. M., Filgueiras-Rama D. (2019). In Vivo Ratiometric Optical Mapping
Enables High-Resolution Cardiac Electrophysiology in Pig Models. Cardiovasc. Res..

[20] Baark F., Shaughnessy F., Pell V. R., Clark J. E., Eykyn T. R., Blower P., Southworth R. (2019). Tissue Acidosis Does Not Mediate
the Hypoxia Selectivity of [64Cu]­[Cu­(ATSM)] in the Isolated Perfused
Rat Heart. Sci. Rep..

[21] Wallabregue A. L., Bolland H., Faulkner S., Hammond E. M., Conway S. J. (2023). Two Color
Imaging of Different Hypoxia Levels in Cancer Cells. J. Am. Chem. Soc..

[22] Perry S. W., Norman J. P., Barbieri J., Brown E. B., Gelbard H. A. (2011). Mitochondrial
Membrane Potential Probes and the Proton Gradient: A Practical Usage
Guide. BioTechniques.

[23] Lagarto J., Dyer B. T., Talbot C., Sikkel M. B., Peters N. S., French P. M. W., Lyon A. R., Dunsby C. (2015). Application of Time-Resolved
Autofluorescence to Label-Free in Vivo Optical Mapping of Changes
in Tissue Matrix and Metabolism Associated with Myocardial Infarction
and Heart Failure. Biomed. Opt. Express.

[24] Mosca S., Dey P., Salimi M., Gardner B., Palombo F., Stone N., Matousek P. (2021). Spatially Offset Raman Spectroscopy—How Deep?. Anal. Chem..

