# Supplementary material for: Multiplexed Fiber-Optic Fluorescence for Functional Monitoring of Perfused Hearts
Source: Anal Chem. 2025 Oct 22;97(43):23896–904. doi: 10.1021/acs.analchem.5c03270 (PMC12590462; doi:10.1021/acs.analchem.5c03270)
Supplement: Supplementary file 2 [file ac5c03270_si_002.pdf]

SUPPLEMENTARY INFORMATION

MULTIPLEXED FIBER-OPTIC FLUORESCENCE FOR FUNCTIONAL MONITORING OF PERFUSED HEARTS

JIANRONG QIU,[1,†] EDWARD WATERS,[2,†] EMILY LUPTON,[2] FRIEDRICH BAARK ,[2] ANTOINE L. D. WALLABREGUE,[3] STUART J. CONWAY,[4] RICHARD SOUTHWORTH[2,*] AND MADS S. BERGHOLT [1,*]

[1]*Centre for Craniofacial and Regenerative Biology, King's College London, London SE1 9RT, UK*

[2]*School of Biomedical Engineering & Imaging Sciences, King's College London, King's Health Partners, St Thomas' Hospital, London SE1 7EH, UK*

[3]*Department of Chemistry, University of Oxford, Oxford OX1 3TA, UK*

[4]*Department of Chemistry & Biochemistry, UCLA, Los Angeles CA 90095-1569, US*

[†]*These authors contributed equally.*

**richard.southworth@kcl.ac.uk, mads.bergholt@kcl.ac.uk*

Table of Contents

**Materials and Methods**	Synthesis of DDAO and Tokyo Green	Page S2, S3
**Figure S1**	Stability of excitation sources recorded at 100 Hz	Page S4
**Figure S2**	Spectrum of unknown autofluorescence	Page S4

Materials and Methods

Fluorophore synthesis

Chemicals were obtained from Sigma-Aldrich UK, Alfa Aesar UK, and Fluorochem, and were used without further purification unless otherwise specified. Brine refers to a saturated aqueous solution of sodium chloride. Anhydrous solvents were obtained from an MBRAUN Solvent Purification System 5 and stored under an argon atmosphere over 3 Å molecular sieves. Petroleum ether refers to the fraction of light petroleum ether boiling in the range 40–60 °C. *In vacuo* refers to the removal of solvent using a Buchi® rotary evaporator under reduced pressure in a water bath at 40 °C.

3-(2'-Hydroxypropan-2'-yl)phenol

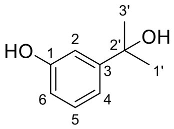

Methyl magnesium chloride (40.0 mL, 119 mmol, 2.2 eq., 3 M in diethyl ether) was added dropwise to a solution of 1-(3-hydroxyphenyl)ethanone (7.4 g, 54.0 mmol, 1.0 eq.) in anhydrous tetrahydrofuran (300 mL) at 0 °C under an argon atmosphere. The reaction mixture was stirred at room temperature for 18 hours and then heated under reflux for 2 hours. After this time the reaction mixture was cooled to 0 °C and saturated aqueous ammonium chloride (50 mL) was added, followed by aqueous hydrochloric acid solution (120 mL, 1 M solution). The aqueous phase was extracted with ethyl acetate (2 × 150 mL). The combined organic components were washed with brine (2 × 200 mL), dried (sodium sulfate), filtered, and concentrated *in vacuo*. The crude material was purified using silica gel column chromatography eluting with ethyl acetate:petrol ether (gradient 10% to 25% ethyl acetate) to give the title compound as a pale-yellow solid (3.6 g, 44%): R_f 0.35 (petroleum ether/ethyl acetate, 1:1); m.p. 99–101 °C (from ethyl acetate). ^{1}H NMR (400 MHz, $CDCl_3$) δ 7.21 (dd, *J* = 7.8, 0.6 Hz, 1H, *H-4*), 7.06–6.98 (m, 2H, *H-2* and *H-6*), 6.72 (ddd, *J* = 7.8, 2.5, 0.9 Hz, 1H, *H-5*), 5.09 (s, 1H, O*H*), 1.57 (s, 6H, *H-3'* and *H-1'*); LRMS *m/z* (ESI^+ not detected). The spectroscopic data are in good agreement with literature values[1].

1,3-Dichloro-7-hydroxy-9,9-dimethylacridin-2(9*H*)-one (DDAO)

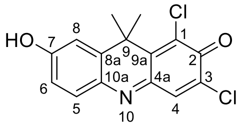

Aqueous sodium hydroxide (21.0 mL, 42.0 mmol, 2.1 eq. 2 M solution) was added dropwise to a solution of 3-(2'-Hydroxypropan-2'-yl)phenol (3.04 g, 20.0 mmol, 1.0 eq.) and 2,6-dichloroquinone-4-chloroimide (4.21 g, 20.0 mmol, 1.0 eq.) in tetrahydrofuran (10 mL) and water (10 mL) at 0 °C. The mixture was stirred for 2 hours, then saturated aqueous ammonium chloride solution (700 mL) was added to the reaction mixture. The aqueous phase was extracted with ethyl acetate (4 × 200 mL). The combined organic components were washed with 10% *w/v* aqueous sodium dithionite solution (2 × 200 mL) and brine (2 × 200 mL). After evaporation of ethyl acetate, the residue was dissolved into methanol (40 mL) and mixed into deoxygenated aqueous hydrochloric acid (500 mL, 2 M solution). The resulting suspension was heated under reflux, under an argon atmosphere for 1.5 hours, during which time a gummy solid formed. The mixture was cooled to room temperature, extracted with ethyl acetate (2 × 400 mL), and the combined organic components were washed with brine (100 mL). A solution of sodium periodate (5.99 g, 28.0 mmol, 1.4 eq.) in water (200 mL) was added to the combined organic components and the resulting solution was stirred vigorously for 16 hours. After this time the aqueous and organic components were separated, and the organic component was washed with brine (200 mL), dried (sodium sulfate), filtered, and

concentrated *in vacuo*. The residue was taken up in hot ethanol (800 mL) and concentrated to *ca*. 150 mL by boiling. Upon cooling, the product was filtered by suction and the black solid was washed with cold ethanol (3 × 50 mL). The solid was dried under vacuum to give the title compound as black needles (5.3 g, 86%): R_f 0.5 (dichloromethane/methanol, 95:5); m.p. >290 °C (from ethanol); ^{1}H NMR (400 MHz, D_6-DMSO) δ 7.78 (s, 1H, *H-4*), 7.51 (d, *J* = 8.6 Hz, 1H, *H-5*), 7.06 (d, *J* = 2.3 Hz, 1H, *H-8*), 6.85 (dd, *J* = 8.7, 2.2 Hz, 1H, *H-6*), 1.79 (s, 6H, 2 × CH_3); LRMS: *m/z* (ESI$^+$) 308.751 ([M+H]$^+$, 26.71%). Analytical HPLC (Dionex Acclaim™ 120 C18 column [5 µm, 120 Å, 4.6 × 150 mm]; 95:5 H_2O: MeCN → 5:95 H_2O: MeCN: H_2O with 0.1% TFA modifier, 10 min; 5 min hold; 1.5 mL min^{-1}] Ret. Time = 10.5 min. Purity: 100%. The spectroscopic data are in good agreement with literature values[1].

6-Hydroxy-9-(2'-methylphenyl)-3*H*-xanthen-3-one (Tokyo green)

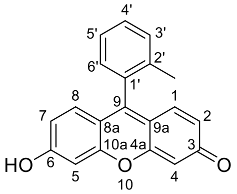

3,6-Dihydroxy-9*H*-xanthen-9-one (0.50 g, 2.19 mmol, 1.0 eq.) was dissolved in anhydrous *N*,*N*-dimethylformamide (45 mL) and to this was added *tert*-butyldimethylsilyl chloride (1.98 g, 13.1 mmol, 6.0 eq.) and imidazole (1.49 g, 21.9 mmol, 10 eq.). After stirring at room temperature for 4 hours, the reaction mixture was diluted with toluene (100 mL), washed with water (5 × 100 mL), dried (sodium sulfate), and filtered. Concentration *in vacuo* gave 3,6-bis((*tert*-butyldimethylsilyl) oxy)-9*H*-xanthen-9-one as an off-white solid (1.00 g) which was used without further purification.

O-Tolyl magnesium bromide solution (1.82 mL, 3.65 mmol, 1.5 eq.) was cooled to 0 °C, then (6.0 mL, 0.30 mmol) of 3,6-bis((*tert*-butyldimethylsilyl) oxy)-9*H*-xanthen-9-one in anhydrous tetrahydrofuran was added, and the mixture was stirred for 25 minutes. After this time aqueous hydrochloric acid solution (95 mL, 2 M solution) was added, and the suspension was stirred for 30 minutes. The resulting yellow precipitate was collected by filtration, washed with aqueous hydrochloric acid solution (4 × 25 mL, 2 M solution), cold tetrahydrofuran (2 × 50 mL), and concentrated *in vacuo*. The crude material was purified using silica gel column chromatography eluting with dichloromethane:methanol (gradient 0 to 10% methanol) to give the title compound as an orange solid (648 mg, 88%): R_f 0.18 (dichloromethane:methanol, 95:5); m.p. 225–227 °C (from water); ^{1}H NMR (400 MHz, D_6-DMSO) δ 7.60 (dd, *J* = 7.5, 1.4 Hz, 1H, *H-6'*), 7.56 – 7.53 (m, 1H, *H-4'*), 7.49 – 7.48 (m 1H, *H-5'*), 7.37 – 7.16 (m, 5H, *H-1, H-5, H-7, H-8 & H-3'*), 7.16 – 7.08 (m, 2H, *H-2 & H-4*), 2.01 (s, 3H, CH_3); LRMS *m/z* (ESI$^+$) 303 ([M+H]$^+$, 100%). Analytical HPLC: Dionex Acclaim® 120 C18 column [5 µm, 12 Å, 150 mm × 4.6 mm]; [95:5 H_2O: MeCN → 5:95 H_2O: MeCN: H_2O with 0.1% TFA modifier, 10 min; 5 min hold; 1.5 mLmin^{-1}] Ret. Time = 7.8 min. Purity: 98.4%. The spectroscopic data are in good agreement with literature values[1].

Stability of excitation sources at 100 Hz

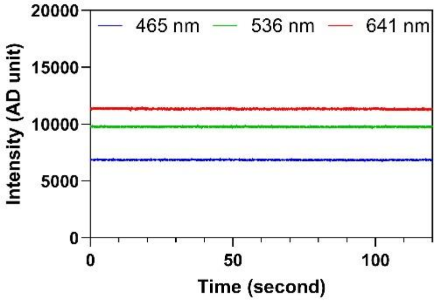

Figure S1. Stability of excitation sources recorded at 100 Hz shows minimal intensity noise, confirming the capacity to monitor rapid biochemical processes. The intensities were measured by the spectrometer, with its input connected to the output of the fiber probe via an optical attenuator. The root-mean-square (RMS) noise levels are 30.6 analog-to-digital units (ADUs), 35.9 ADUs, and 41.7 ADUs at the wavelengths of 465 nm, 536 nm and 641 nm respectively.

Spectrum of unknown autofluorescence

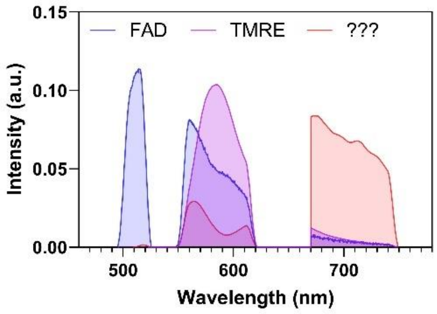

Figure S2. Emission spectra of FAD, TMRE and the unknown autofluorescence.

References

1. Wallabregue, A. L.; Bolland, H.; Faulkner, S.; Hammond, E. M.; Conway, S. J. Two Color Imaging of Different Hypoxia Levels in Cancer Cells. *J. Am. Chem. Soc.* **2023**, *145* (4), 2572–2583.
